# ISL1 Directly Regulates *FGF10* Transcription during Human Cardiac Outflow Formation

**DOI:** 10.1371/journal.pone.0030677

**Published:** 2012-01-27

**Authors:** Christelle Golzio, Emmanuelle Havis, Philippe Daubas, Gregory Nuel, Candice Babarit, Arnold Munnich, Michel Vekemans, Stéphane Zaffran, Stanislas Lyonnet, Heather C. Etchevers

**Affiliations:** 1 Center for Human Disease Modeling, Department of Cell Biology, Duke Medical Center, Durham, North Carolina, United States of America; 2 UPMC Univ Paris 06, CNRS UMR 7622, Paris, France; 3 CNRS URA 2578, Institut Pasteur, Paris, France; 4 CNRS 8145, Mathématiques appliquées, Université Paris Descartes, Paris, France; 5 INSERM U781, Université Paris Descartes, Faculté de Médecine, Paris, France; 6 Service de Génétique Médicale, Hôpital Necker-Enfants Malades, Paris, France; 7 INSERM, U910, Marseille, France; Aix-Marseille Univ, Faculté de Médecine, UMR 910, Marseille, France; Ecole Normale Supérieure de Lyon, France

## Abstract

The LIM homeodomain gene Islet-1 (*ISL1*) encodes a transcription factor that has been associated with the multipotency of human cardiac progenitors, and in mice enables the correct deployment of second heart field (SHF) cells to become the myocardium of atria, right ventricle and outflow tract. Other markers have been identified that characterize subdomains of the SHF, such as the fibroblast growth factor *Fgf10* in its anterior region. While functional evidence of its essential contribution has been demonstrated in many vertebrate species, SHF expression of *Isl1* has been shown in only some models. We examined the relationship between human ISL1 and FGF10 within the embryonic time window during which the linear heart tube remodels into four chambers. *ISL1* transcription demarcated an anatomical region supporting the conserved existence of a SHF in humans, and transcription factors of the GATA family were co-expressed therein. In conjunction, we identified a novel enhancer containing a highly conserved ISL1 consensus binding site within the *FGF10* first intron. ChIP and EMSA demonstrated its direct occupation by ISL1. Transcription mediated by ISL1 from this *FGF10* intronic element was enhanced by the presence of GATA4 and TBX20 cardiac transcription factors. Finally, transgenic mice confirmed that endogenous factors bound the human *FGF10* intronic enhancer to drive reporter expression in the developing cardiac outflow tract. These findings highlight the interest of examining developmental regulatory networks directly in human tissues, when possible, to assess candidate non-coding regions that may be responsible for congenital malformations.

## Introduction

Congenital heart malformations occur in approximately 3 per 1000 births, more than half of which are potentially lethal malformations of the outflow tract (OFT) [1]. Extensive studies have been undertaken to identify factors driving the differentiation of cell populations that participate in OFT formation in mice and other species, with the expectation that functional data about evolutionarily conserved molecules can be extrapolated to human development.

Two spatially distinct groups of myocardial progenitors, located in the first and the second heart fields, contribute to the definitive heart pump [2], [3]. The chambers proper are derived from the former, while the outflow segment of the right ventricle and great arteries and the inflow portion of the atria come from the latter. Initially identified in mouse and chick embryos, there appears to be equivalent spatial segregation between progenitor lineages in lower vertebrates without four-chambered hearts, recently identified in frog [4] and fish [5].

Coordination between these separate but adjacent mesodermal primordia is orchestrated by signaling events that converge on a common palette of transcription factors necessary for the site-appropriate differentiation of the multiple cell types present in a mature heart. The LIM homeodomain transcription factor Islet-1 (Isl1) is one of these. *Isl1* is necessary for multipotent cardiovascular progenitors within the second heart field to proliferate, survive, and migrate into the forming heart. Isl1 is highly conserved over chordate evolution in this role [4], [6]. *Isl1*-null mice die at mid-gestation from gross cardiac malformations, notably the lack of the OFT and right ventricle myocardium [7]. Isl1 is also known to be critical for formation and specification of motoneurons [8] and of the pancreas [9], acting in combination with other transcription factors to attain specific and context-dependent effects on differentiation [8].

In the developing heart, these combinatorial partners include members of the tinman (Nkx), GATA-binding and T-box (Tbx) families [10]–[12], which may derepress and add permissive marks to chromatin [13]. Such associations indeed appear to be stabilized by the preparatory activity of Swi/Snf-like BAF chromatin remodelling complexes expressed precisely within heart precursor primordia, such as Smarcd3 (Baf60c) [14].

For example, murine Isl1 directly controls the expression of the early mesodermal transcription factors *Mef2c* and *Nkx2-5* during cardiac development via elements in their promoters that also contain nearby, active GATA-binding sites [10], [15]. In return, human NKX2-5 itself can bind the *GATA4* promoter to positively control its transcription during fetal cardiomyocyte differentiation [16], while forced co-expression of Smarcd3, Gata4 and Tbx5 can induce *Isl1* and *Nkx2.5* expression in murine mesoderm not normally fated to integrate the heart, leading to cardiac transdifferentiation [17].

No *ISL1* coding mutations have been identified in humans, probably because of an embryonic lethal phenotype for complete inactivation and no gross effect of haploinsufficiency, as seen for murine *Isl1* [7]. Heterozygous *ISL1* mutations have not directly been reported to cause conotruncal cardiopathies either, although a block of single nucleotide polymorphisms around and within *ISL1* have indeed been found to be in linkage disequilibrium with a risk for complex congenital heart phenotypes involving “developmental structures aberrantly formed as derivatives of the secondary [sic] heart field.” [18].

In *Isl1* homozygous knockout mice, the residual hearts no longer express certain bone morphogenetic protein (Bmp) or Wnt family members, Fgf8 or Fgf10, and are missing the OFT entirely [7]. Fgf10, a secreted member of the fibroblast growth factor family, also characterizes the splanchnic mesoderm of the anterior majority of the murine second heart field [3]. In the mouse, its genetic ablation leads to absence of pulmonary arteries and veins, malposition of the heart apex and thin-walled myocardium [19], [20]; the absence of the cognate specific receptor isoform for Fgf10, Fgfr2-IIIb, leads in knockout mice to pulmonary vessel aplasia and to OFT malformations such as double outlet right ventricle or ventricular septal defects with overriding aorta [19]. Despite its strong and specific expression in the murine OFT, the function of cardiac Fgf10 has been difficult to ascertain, and its direct transcriptional regulation by Isl1 suggested but not demonstrated in this tissue. Only Tbx1 and Tbx5 have so far been shown to directly bind to and positively regulate *Fgf10* expression in the OFT through a 5′ enhancer element [21], [22]. However, Isl1 and Fgf10 also play early roles in the specification and outgrowth of vertebrate hindlimbs [23]–[25], while a consensus Isl1-binding site was identified *in silico* within a 0.4 kb *Fgf10* promoter element that is highly conserved among amniotes and capable of directing expression to the otic anlage [26].

The phenotype of *Fgf10*-null mice demonstrates the irreplaceable role of Fgf10 in epithelial-mesenchymal interactions needed for the development of many organ systems, including but not restricted to endodermal organs and glands of the head and neck [24], [27], [28]. However, there appears to be partial functional redundancy with other Fgf family members, including Fgf3 and Fgf8, in the heart and great vessels [29]–[31], and different Fgfs in other organ systems such as the inner ear, pituitary and limb buds [32], [33]. Human heterozygous mutations of *FGF10* lead to isolated or syndromic aplasia of the lacrimal and salivary glands and ducts [34], [35], not clearly involving the heart, hindgut, ear, pancreas or limbs, that were severely affected in homozygous knockout mice but less so or not at all in heterozygotes. The effect on the lungs is subtle and cumulative in haploinsufficient patients, leading to chronic obstructive pulmonary disease [36]. Like for *ISL1*, no biallelic inactivation of *FGF10* has been found to date in human disease [37], but the more subtle effects of *Fgf10* ^+/−^ phenotypes have only been described progressively over the years since the first murine knockout models.

The spatiotemporal expression of human *ISL1* has recently been demonstrated to be compatible with the existence of a subset of embryonic progenitors that would contribute specifically to the inflow and outflow tracts, as in animal models [11], or that maintain developmental plasticity at later fetal stages [38]. In this work, we demonstrate not only that *ISL1* is co-expressed with other transcription factors in the cardiac primordium, but that *in vivo* it directly binds and positively regulates the transcription of *FGF10*. ISL1 exerts this effect through an enhancer within the *FGF10* first intron that is evolutionarily conserved among mammals, becomes additionally responsive to ISL1 *in vitro* in the presence of GATA and TBX factors, and is capable of responding to endogenous cardiac OFT transcription factors in a transgenic mouse reporter.

## Results

### ISL1 binds a novel intronic element of the *FGF10* gene in the human heart but not hindlimb

**Figure 1:**
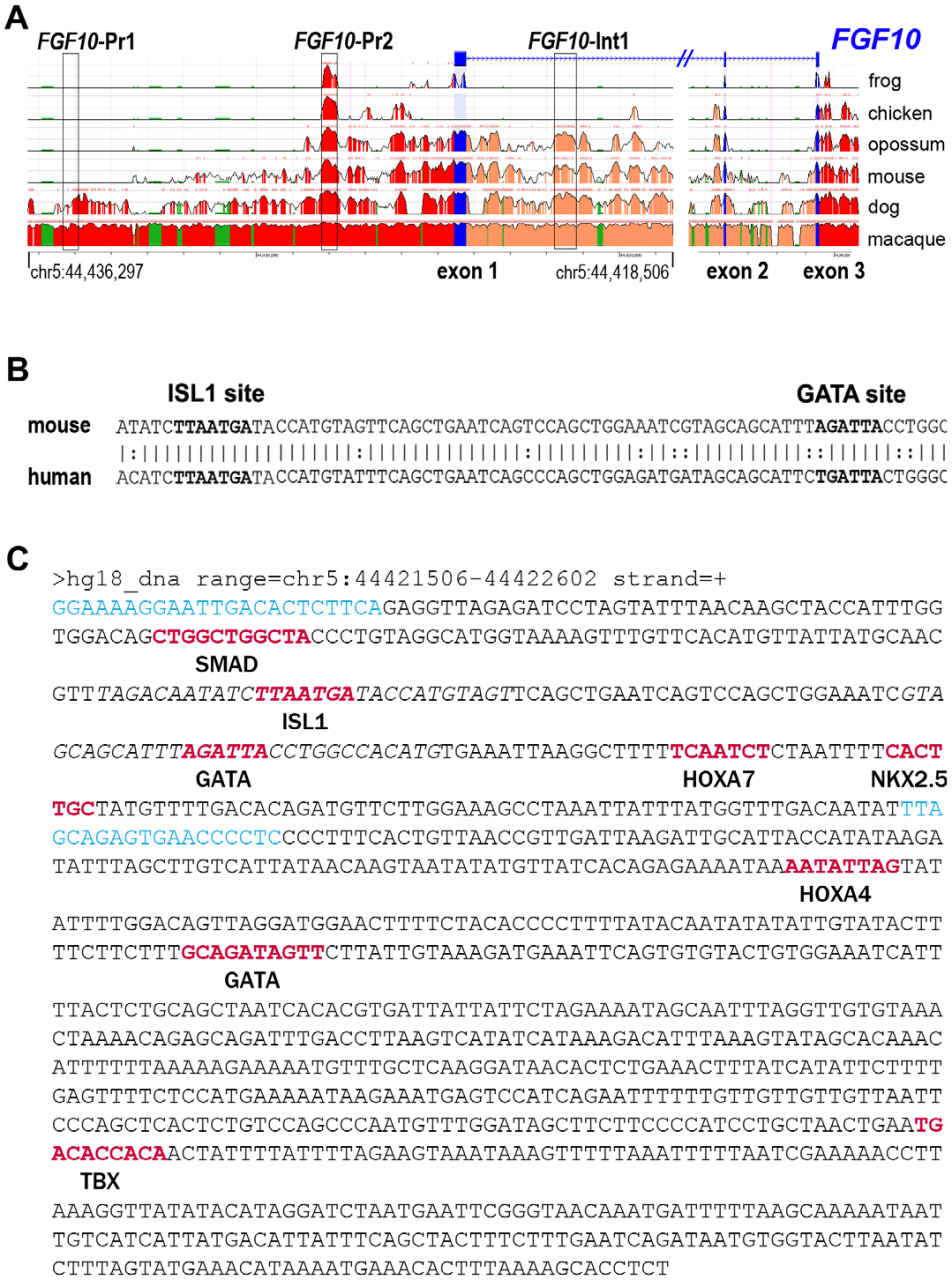
Bioinformatics analyses of the human *FGF10* locus surrounding the first exon. **A**: Alignment of genomic regions around and within the human [hg18] *FGF10* locus to those of frog [xenTro2], chicken [galGal3], opossum [monDom4], mouse [mm9], dog [canFam2] and rhesus macaque [rheMac2] with colored regions >90% identical and the vertical scale ranging from 50% (bottom) to 100% (top). Color code for genomic features at http://ecrbrowser.dcode.org/ecrInstructions/ecrInstructions.html. The *FGF10*-Pr1, *FGF10*-Pr2 and FGF10-Int1 regions examined in this study are boxed. **B**: A non-canonical predicted site for GATA-type transcription factors is 52 nucleotides 5′ to the ISL1 cognate sequence in *FGF10*-Int1 in the direction of transcription on the – strand in humans, mice and (not shown) macaque and opossum. **C**: Nucleotide sequence of the *FGF10*-Int1 enhancer module and position of conserved putative transcription factor binding sites as predicted by rVista (http://rvista.dcode.org). All indicated human sites are identical to those of the macaque and mouse except for the SMAD prediction, only found in mouse; the ISL1, GATA and HOXA7 sites are also identical to the opossum, and the ISL1, NKX2-5 and TBX sites are also identical to the dog.

Recent results from our and other groups have demonstrated the expression of both *FGF10* and *ISL1* in a region probably corresponding to a second heart field in human embryos at appropriate and similar stages of morphogenesis [11], [37]. A non-exhaustive bioinformatics analysis of the *FGF10* locus to search for putative highly conserved ISL1 consensus binding sites with the sequence YTAATGR, using rVista 2.0 (http://rvista.dcode.org) [39] and the ECR browser (http://ecrbrowser.dcode.org) [40], identified two candidate regions conserved among therian mammals (Fig. 1A). One had been previously predicted within the *FGF10* promoter [26] and was also common to birds and amphibians, which we termed *FGF10*-Pr2; another, within the first intron of *FGF10*, was termed *FGF10*-Int1. A third promoter region, without an ISL1 consensus binding site, was designated as *FGF10*-Pr1. A non-canonical (i.e. 5′-TGATTA-3′) potential binding site for GATA-type transcription factors [41] was observed 52 nucleotides 5′ to the ISL1 cognate sequence in *FGF10*-Int1 and these sites were nearly identical in nucleotide composition and distance from one another between mice and humans (Fig. 1B). This attracted our attention to three additional potential sites for homeobox-containing transcription factors and another GATA site, as well as a putative, but less conserved, canonical T-box (Fig. 1C), making all of *FGF10*-Int1 a candidate cis-regulatory module [42]. All other sites identified, within evolutionarily conserved modules, were 100% identical between species.

**Figure 2:**
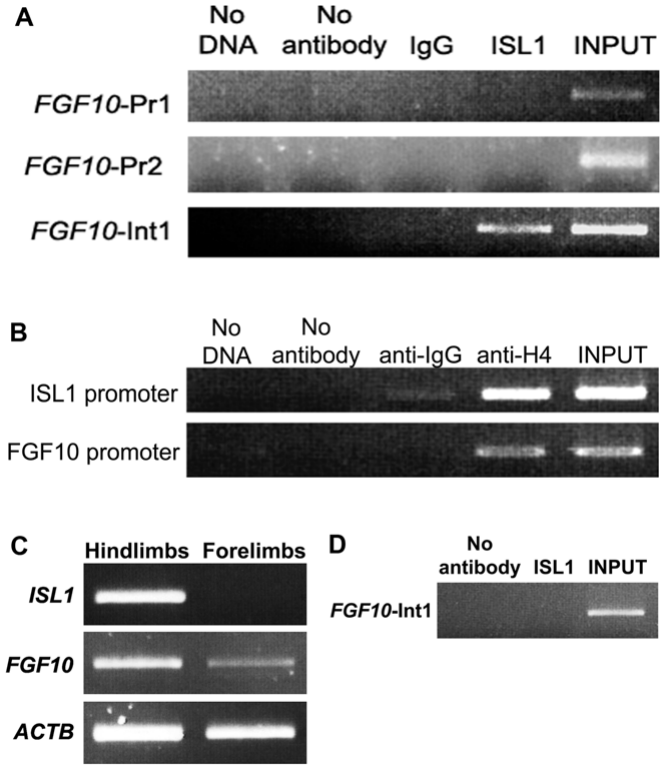
*In vivo* and *in vitro* binding of ISL1 and GATA4 within the first intron of *FGF10*. **A**: Results of end-point PCR after ChIP using anti-ISL1 or non-specific IgG (or no antibody at all) on chromatin derived from human embryonic hearts at Carnegie stages (CS)14-15. **B**: Analogous results using anti-acetylated histone H4 compared to a non-specific IgG or no antibody at all, and end-point PCR of regions in the 5′ promoter to human *ISL1* and *FGF10*, demonstrating active availability for transcription. **C**: *FGF10* and *ISL1* (and *ACTB*) were co-expressed at foot plate stages (Carnegie stages [CS]16-17, i.e. 37–43 days of gestation) in human hindlimbs as seen by RT-PCR, while only *FGF10* and *ACTB* were transcribed in forelimbs. **D**: ChIP using anti-ISL1 on chromatin derived from the C16–17 hindlimb demonstrates no enrichment of the *FGF10*-Int1 amplicon as compared to the negative control, although this fragment is amplifiable from the total input chromatin.

Using chromatin immunoprecipitation (ChIP) of microdissected embryonic human hearts, we demonstrated that at Carnegie stages 14–15 (33–36 dpf), ISL1 bound to and enriched a 327 bp *FGF10*-Int1 fragment (Fig. 2A). In contrast, ISL1 did not occupy *FGF10*-Pr1 or *FGF10*-Pr2. Acetylated histone H4 did bind both the *ISL1* and *FGF10* promoters at CS14-15, confirming that the chromatin around these two promoters is transcriptionally active in the human heart at these stages (Fig. 2B) [43].

We also examined whether ISL1 could bind to the *FGF10*-Int1 element in developing human hindlimb buds, since *FGF10* and *ISL1* are co-transcribed at foot plate stages at CS16-17 (37–43 dpf; Fig. 2C). While *FGF10*-Int1 was occupied by ISL1 in the CS14-15 heart, ChIP performed on CS16-17 hindlimbs demonstrated no equivalent binding of ISL1 to *FGF10*-Int1 (Fig. 2D).

### *ISL1* and *GATA4/5/6* are transcribed in the same temporal window as *FGF10*

**Figure 3:**
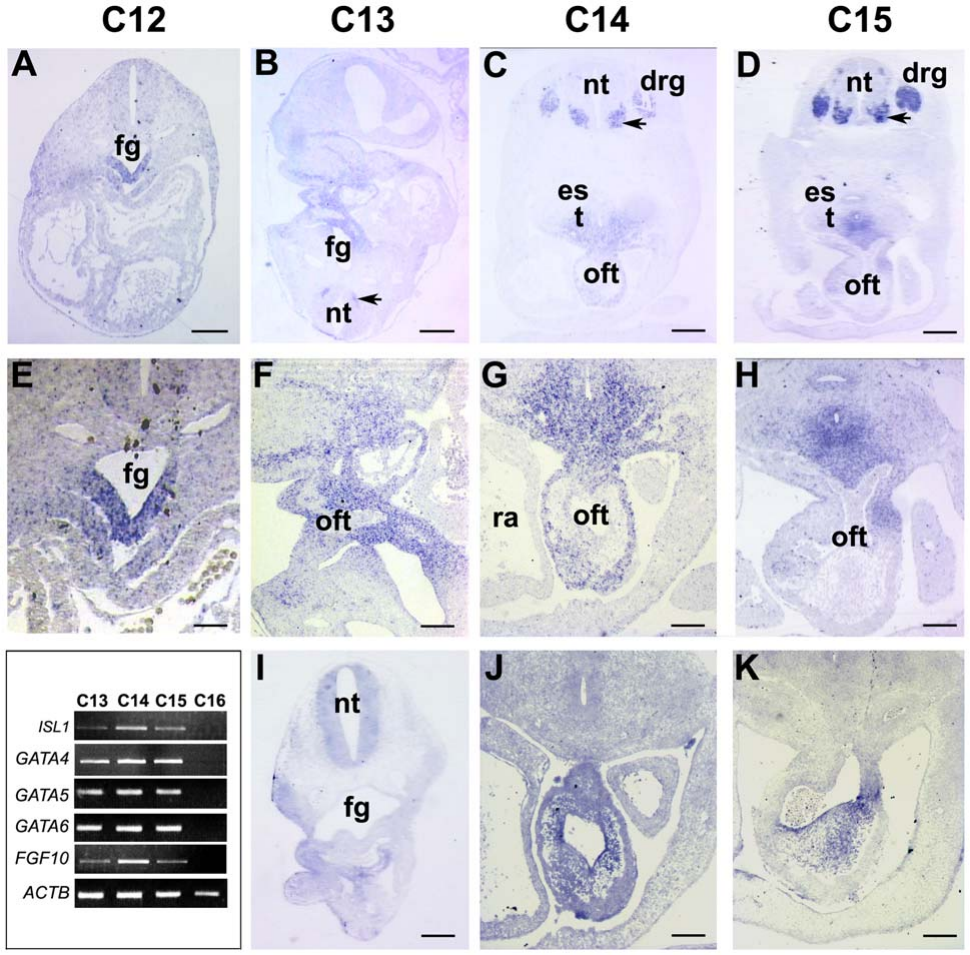
Expression of *ISL1* and *GATA4* transcripts in the human heart between 26 and 38 days of gestation. **A–H**: *ISL1 in situ* at Carnegie stages (CS)12 (26–28 days post fertilization [dpf]), CS13 (28–31 dpf), CS14 (32–33 dpf) and CS15 (34–36 dpf) respectively. **E–H** are magnifications of **A–D** respectively. **I–K** show *GATA4* expression in adjacent sections to **B–D**. **A**: *ISL1* is expressed at CS12 in foregut endoderm, splanchnic mesoderm, and early motoneurons. **B, F**: At CS13, *ISL1* is transcribed by mesenchyme around the cardiac OFT and pharyngeal arches. *ISL1* expression continues in the splanchnic mesoderm between the trachea and OFT, and is visible in dorsal root ganglia, at CS14 (**C, G**) and CS15 (**D, H**). **I–K**: *GATA4* is expressed in the endocardium and myocardium of the arterial pole at CS13, CS14 and CS15 (**I, J, K** respectively). **Inset**: RT-PCR of *ISL1*, *GATA4*, *GATA5*, *GATA6*, *FGF10* and positive control *ACTB* mRNAs in embryonic human hearts at stages CS13-16 (to 40 dpf). Abbreviations: drg, dorsal root ganglia; es, esophagus; fb, forebrain; fg, foregut; ph, pharynx; nt, neural tube; oft, OFT; ra, right atrium; t, trachea. Arrows, motoneurons. Bar: 110 µm (A–D, I) and 55 µm (E–H, J, K).

In light of the presence of putative conserved GATA-binding sites in *FGF10*-Int1, we examined the expression of potential cardiac GATA partners and compared it to that of *ISL1* at a range of stages covering the morphogenetic changes from directional S-shaped looping of the primitive cardiac tube to the appearance of four distinct chambers [44] (Figure S1). RT-PCR of mRNAs extracted from microdissected, staged human heart primordia demonstrated that *ISL1*, *GATA4*, *GATA5*, *GATA6*, and *FGF10*, were all expressed at CS13-15 (28–36 dpf). In contrast, these genes were no longer transcribed at CS16 (37–40 dpf), despite continued expression of the ubiquitous *ACTB* (Fig. 3 inset).

On sections through the heart at CS12, no *GATA4* expression was observed in the outflow tract region, while *ISL1* hybridization was only visible in the endoderm of the ventral foregut (Fig. 3A). In agreement with the RT-PCR data, at CS13-15 both *ISL1* and *GATA4* were transcribed within the cardiac mesenchyme surrounding the aorticopulmonary trunk (see magnifications Fig. 3F–H and I–K, respectively). *ISL1* also maintained expression at CS15 within the splanchic mesenchyme between the trachea and the heart, while *GATA4* appeared restricted to the endocardium and myocardium at CS14-15.

### ISL1 and GATA4 each can bind the *FGF10-Int1* element *in vitro*

**Figure 4:**
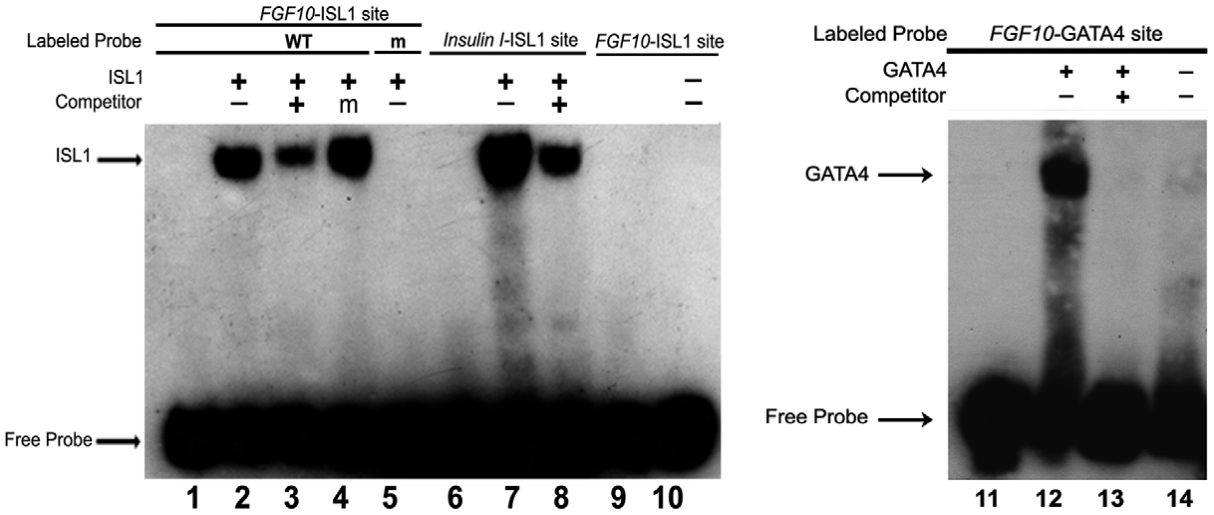
Electrophoretic mobility shift assays demonstrate specific binding of both ISL1 and GATA4 to the conserved *FGF10* intronic element (*FGF10*-Int1). Lane 1, 9, 10: WT *FGF10*-ISL1 site probe alone, or in conjunction with ISL1 (lane 2) and with unlabeled competitor, which reduces the amount of shifted probe (lane 3), or with ISL1 and unlabeled competitor carrying a mutation in the ISL1 binding site (lane 4). Mutated ISL1 does not shift this probe (lane 5). Lane 6: A validated tandem set of ISL1 binding sites from the insulin promoter shows no gel shift unless ISL1 is added (lane 7) and this shift is reduced in the presence of unlabelled probe competitor (lane 8). Lane 11, 14: WT FGF10-GATA site probe alone, or in conjunction with GATA4 (lane 12) and with unlabeled competitor, which completely abrogates the shift of the probe (lane 13).

To investigate the specificity of ISL1 binding to its consensus site within *FGF10*-Int1, we performed an electrophoretic mobility shift assay (EMSA, Fig. 4). ISL1 bound robustly to its *FGF10*-ISL1 site, as well as to a previously identified positive control site [10], termed *Insulin I*-ISL1 (Fig. 4, lanes 2 and 7 respectively). The *FGF10*-ISL1 binding was specific, since it could be partially competed off by excess unlabeled probe (Fig. 4, lane 3) but not by a hundredfold excess of unlabeled mutated probe (Fig. 4, lane 4). In addition, ISL1 did not bind to a labeled, scrambled *FGF10*-ISL1 sequence (Fig. 4, lane 5).

In order to verify the affinity of the nearby, non-canonical GATA site in *FGF10*-Int1 for GATA4, we performed another EMSA, confirming that GATA4 was able to occupy this sequence (Fig. 4, lane 12). Binding to the 5′-TGATTA-3′ site was completely abrogated by the addition of unlabeled *FGF10*-GATA4 probe (Fig. 4, lane 13).

### ISL1 and GATA4 cooperate with TBX20 to activate *FGF10* via its intronic enhancer

**Figure 5:**
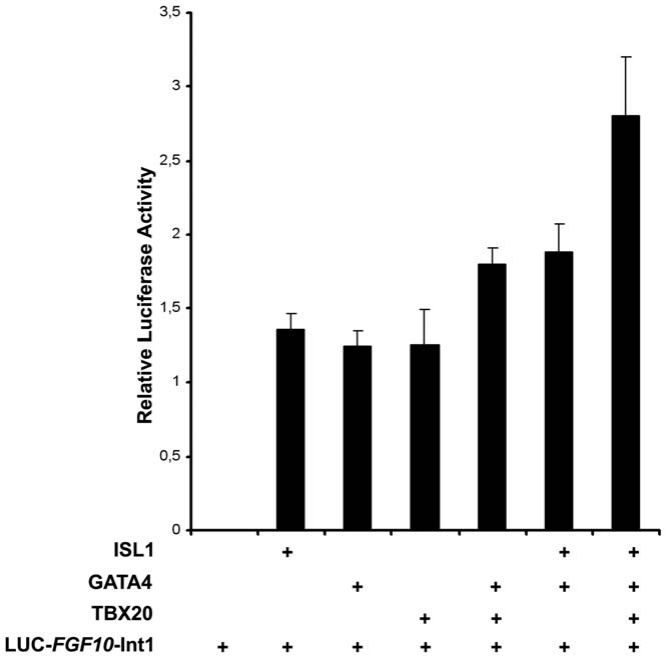
*In vitro* reporter assays support an additive combinatorial effect of transcription factors upon the FGF10 intronic enhancer. LUC-*FGF10*-Int1, which construct placed the luciferase gene under the control of the FGF10-Int1 element, was transfected alone or together with *ISL1*, *GATA4* and *TBX20* expression vectors into 10T1/2 cells. Each factor alone potentiated luciferase expression and these effects were additive in combination.

The transcriptional response of murine *Nkx2.5* to the combination of Isl1 and Gata4 *in vitro* can be potentiated by Tbx20, a member of a large family of genes whose products share a common DNA-binding domain, similar to the *T* (brachyury) transcription factor [15]. We first determined the ability of ISL1 and/or GATA4 to promote luciferase activity using a reporter with a minimal promoter containing the human cardiac-responsive *FGF10-Int1* fragment located 3′ to the luciferase sequence, mimicking the endogenous location of this regulatory element relative to the initiation site for *FGF10* transcription. Co-transfection into mesenchymal 10T1/2 cells of a GATA4 or ISL1 expression construct, together with the *FGF10*-Int1-luciferase reporter, indeed resulted in robust activation of luciferase activity (Fig. 5).

We then tested whether this human *FGF10*-Int1 element could drive expression of a luciferase reporter gene in the presence of ISL1, GATA4, and TBX20 proteins separately as well as in combination. Despite the presence of only a single, non-palindromic T-box binding core motif [45] within the intronic response element (Fig. 1), transfection of TBX20 in addition to GATA4 and ISL1 expression constructs resulted in additive activation of *FGF10*-Int1-luc (Fig. 5).

### Transgenic mouse embryos express *FGF10-Int1*-driven reporter in cardiac OFT

The strict sequence conservation between humans and mice, and the ability of transfected murine cells to demonstrate ISL1- and cofactor-driven activation of a reporter gene containing the *FGF10*-Int1 enhancer *in vitro*, led us to then test the ability of the element to drive reporter expression when introduced *in vivo*. *FGF10*-Int1 was therefore subcloned into the pTK-nlacZ reporter plasmid [46] and introduced into mouse blastocysts. 43 embryos out of 66 injected were recovered at E8.5, 22 of 53 at E9.5, 46 of 94 at E10.5, and 37 of 59 at E11.5. Of these, nine animals had integrated the transgene, confirmed by PCR, and expressed beta-galactosidase activity: n = 2 at E8.5, n = 3 at E9.5, n = 1 at E10.5 and n = 3 at E11.5.

**Figure 6:**
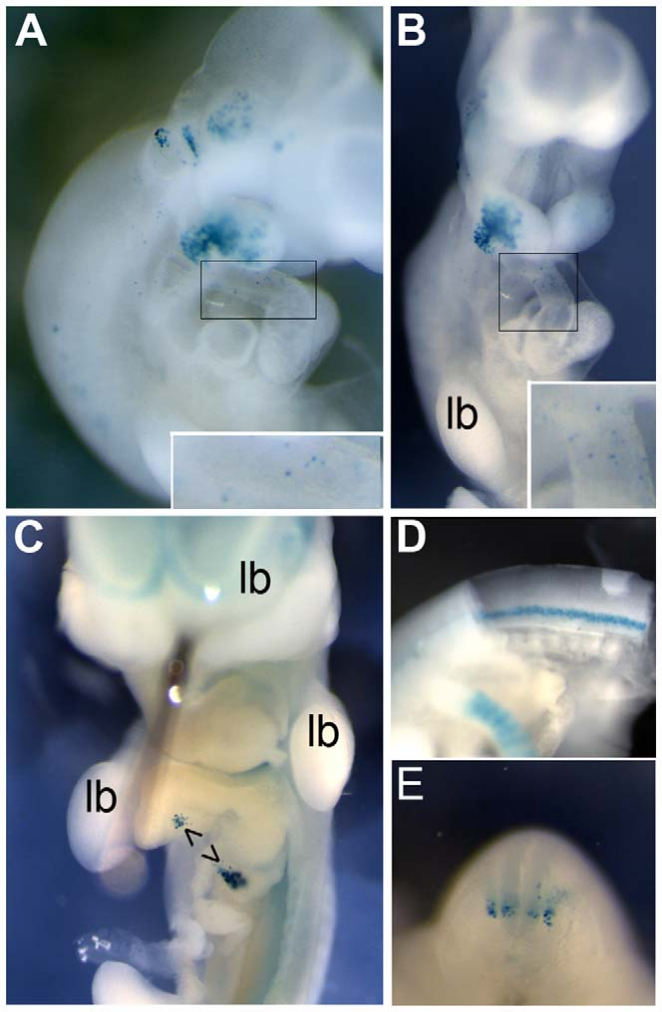
Transgenic mice demonstrate responsiveness of the conserved *FGF10* intronic enhancer to endogenous transcription factors within the developing cardiac OFT and other sites. A 1047 bp enhancer region within the first intron of human *FGF10*, containing multiple transcription factor binding sites including sites validated for ISL1 and GATA4, was placed ahead of a *lacZ* reporter gene under a thymidine kinase-driven promoter. **A**: Transgenic mouse at embryonic day (E)10.5, in which expression was activated in dispersed cells of the posterior outflow tract (magnified, insets), in a distal/lateral subdomain of the first two pharyngeal arches, in cells within the trigeminal, acoustic and dorsal root ganglia, and in the lens (right side). **B**: Same embryo; frontal view. **C**: Transgenic mouse at E11.5, dorsal and ventral pancreatic primordia. No expression was observed in the limb buds in any injected embryos. In a different transgenic mouse at E11.5, **D**: motoneuron columns from inner surface of the lumbar spinal cord, and **E**: cross-section of spinal cord with a labelled subpopulation of cells in the dorsal root ganglia.

Labelled cells were observed in the cardiac outflow tract in two of the reporter embryos, at E9.5 and E10.5 respectively, demonstrating the conserved ability of this enhancer to drive gene transcription in both mouse and human hearts. Expression in both cases concerned a few dozen cells, which were not observed in other heart compartments (Fig. 6A–B). Among the positive embryos, a restricted set of additional tissues were also labelled, varying in combinations from one embryo to another in an age-appropriate manner (Table 1). These included the forebrain, the lens, the three first pharyngeal arches, the pancreatic primordia (dorsal and ventral; Fig. 6C), a subset of dorsal root ganglia cells, and motoneurons (Fig. 6D–E). Scattered cells were also positive in the rostral presomitic mesoderm in both E8.5 embryos. Although neither cardiac nor pharyngeal arch expression were visible in the three E11.5 embryos, the *tunica media* of the internal carotid arteries were positive in one, and the trigeminal and acoustic ganglia were labelled in another. Overall, the sites of transgenic labelling are compatible with activation by Isl1, given what is known about its expression pattern in all of these sites at these stages of development [7], [9], [47], [48], and thus with its positive regulation of *FGF10* transcription in both the human and murine cardiac OFT.

**Table 1:** Sites of β-galactosidase activity in transgenic mouse embryos.

Age	forebrain	lens	MNs	DRGs	pancreas	PSM	PA1	PA2	PA3	OFT
E8.5	−	n/a	.	n/a	n/a	+	.	n/a	n/a	.
E8.5	+	n/a	.	n/a	n/a	+	.	n/a	n/a	.
E9.5	.	n/a	.	.	.	−	+	+	.	.
E9.5	.	n/a	.	.	.	−	.	+	.	+
E9.5	.	n/a	.	.	+	−	+	+	.	.
E10.5	+	+	.	+	.	−	+	+	+	+
E11.5	+	+	+	+	+	−	.	.	+	.
E11.5	+	+	+	+	.	−	.	.	.	.
E11.5	+		+	+	.	−	.	.	.	.

All sites showed only selective cells positive for enhancer activation. DRGs = dorsal root ganglia; E = embryonic day of gestation; MN = motoneurons; OFT = cardiac outflow tract; PA = pharyngeal arch; PSM = pre-somitic mesoderm.

## Discussion

We have found that within the first intron of the *FGF10* gene there exist highly evolutionarily conserved consensus binding sites for equally conserved transcription factors of the LIM homeodomain, GATA and T box families. These sites are arranged in such a way as to represent a functional cis-regulatory module, with physical spacing between the binding sites that is itself also conserved across species, in particular that between the ISL1 and GATA cognate sites. We have demonstrated that in the human embryonic heart, this module is physically occupied by ISL1 during the period corresponding to the establishment of the cardiac chambers but before septation of the OFT [44]. Binding of ISL1 to the intronic element of *FGF10* then ceases in the cardiac OFT, but is never observed in the human or mouse hindlimb bud, for example, where both *Isl1* and *Fgf10* are expressed shortly thereafter. This observation shows tissue specificity in the function of this binding site and is consistent with the *ISL1* expression pattern that we and others [11] have observed in the human embryonic OFT as well as in the splanchnic mesoderm between CS13-15, as reported in mouse at equivalent morphological stages [49]. Despite a great deal of study of tissue-specific enhancers engaged by Isl1 [10], [50] and the control of *Fgf10* expression by transcription factors in the limb [51] and inner ear [26], [52], this is the first report of cis-regulation of *FGF10* expression through an intronic element during cardiac development.

*In situ* hybridization to *GATA4* transcripts in adjacent sections demonstrated that at CS12, unlike the morphologically equivalent stage in the mouse [53], no *GATA4* expression was observed in the OFT region. Other subtle differences exist as well between the mouse and human patterns, notably the lack of *ISL1* expression outside of the pharyngeal endoderm at CS12, when in the mouse, it is also strongly expressed in the underlying ventral splanchnic mesoderm from an earlier stage [7]. During murine OFT maturation, *Isl1*- and *Gata4*-expressing cardiac mesenchyme is also colonized by neural crest cells. However, in the mouse, *Isl1* is never expressed in migrating neural crest cells [7], and *Gata4* is rapidly downregulated in both mesectodermal and cardiac neural crest cells [54]. The subpopulation of human *ISL1*-positive cells in the OFT, that apparently also co-expresses *GATA4*, is thus likely to be mesodermal in origin. This localization is compatible with the regulation of *FGF10*.

This conclusion is supported by transgenic mice in which the human *FGF10* response element was introduced to drive transcription of a reporter gene, yielding labeled cells in the OFT at stages that morphologically precede cardiac chamber formation. Our complementary *in vitro* experiments further demonstrated that the single binding site for ISL1 in the 1047 bp *FGF10* response element enriched by ChIP is sufficient to drive a three-fold increase in luciferase activity in response to the presence of ISL1 alone. This represents significant and strong activation, since the reporter construct did not contain tandem ISL1 recognition sites but rather preserved the *in vivo* arrangement of multiple predicted binding sites for conserved transcription factors. Despite the absence of a palindromic T-box consensus site within the intronic response element of *FGF10*, we obtained transactivation of the reporter, which is in accordance with previous studies showing the response of murine *Nkx2.5* to Tbx20 even in the absence of a cognate T-box element [15]. Together with the capacity of GATA4 to transactivate the same reporter in an additive fashion, these results are consistent with a combinatorial action of transcription factors on *FGF10* non-coding elements to confer a state of either permission or transcriptional activation to otherwise refractory chromatin.

Among the many dozens of genes highly conserved through evolution and identified as key effectors of animal cardiogenesis, only a handful of them, including a disproportional number of transcription factors (*GATA4, NKX2.5, ZIC3, TBX1,TBX20 and CHD7* [55]–[60]), but also intracellular effectors (*TAB2* [61], *MID1* [62]) and ligands (*BMP4* [56]) or membrane-bound proteins (*STRA6* [63], [64], *NOTCH1* [65], and *CFC1* [66]), have so far been directly linked to congenital heart malformations of the OFT in humans. Mutations in these genes can lead, infrequently and often in association with other developmental anomalies, to persistent *truncus arteriosus*, double outlet right ventricle, interruption or severe hypoplasia of the aortic arch, tetralogy of Fallot, and valvulopathies. However, there is only partial correspondence between murine and human gene inactivation phenotypes, with many excellent candidate genes through their function in animal model cardiac development not having been found to be mutated in their human counterpart coding sequences.

*FGF10* is one of these latter genes, whose cardiac knockout phenotype in the mouse is itself subtle. Based on the murine phenotypes of *Fgf10* and *Fgfr2-IIIb* knockouts and their expression patterns [3], [19], we had previously found very similar expression during normal human embryonic development; however, sequencing of both *FGF10* and *FGFR2-IIIb* in human fetuses exhibiting great vessel defects that resembled those in knockout mice, among other symptoms, did not demonstrate coding mutations [37]. The responsible gene turned out to encode a protein, STRA6, necessary to bring vitamin A into cells, a first step in transcriptional regulation through retinoic acid receptor binding [63], [64]. Retinoic acid, a vitamin A metabolite, normally favors *Gata4* transcription and limits the spatial expansion of *Isl1*, *Fgf8* and *Fgf10* expression in the SHF [67]–[69], while it promotes *Fgf10* transcription in the burgeoning lungs [70]. Coding mutations in *FGF10* lead to phenotypic defects only in the submandibular and lachrymal glands and lungs [34], [35], despite being as present as *Stra6* [71] in many other organ systems. Similarly, heterozygous missense coding mutations in human *FGF8* have been shown to be associated with non-syndromic cleft lip and palate [72], cause pleiotropic defects in forebrain and pituitary formation [73], and a recent case of recessive holoprosencephaly with asymptomatic, consanguineous parents has been attributed to hypomorphic alleles of *FGF8* [74]; none of these patients presented cardiac malformations. These observations emphasize the danger of extrapolating findings about the detailed mechanisms of action of highly conserved genes across species, and demonstrate the limits of animal models in understanding human organogenesis.

There is increasing evidence that mutations in non-coding, cis-regulatory elements, controlling transcript availability at a given point time or a given tissue, represent an alternative mechanism leading to human congenital malformations. Such mutations can take the forms of those found for coding sequences, involving single nucleotides [75] or small or large chromosomal rearrangements [76]. We have discovered an evolutionarily conserved cis-regulatory module in the *FGF10* gene that is functional during human cardiac development and that could represent an example of the types of non-coding sites in which mutations may be responsible for morphological aberrations. Taken together, our data reveal unexpected complexity in the transcriptional landscape controlling human cardiogenesis, highlight evolutionary conservation as well as species-specific aspects of cardiac signalling networks, and contribute a strategy to identify additional candidate genomic regions for study in congenital malformations of the OFT.

## Materials and Methods

### Ethics statement

Human embryos were obtained from electively terminated pregnancies, anonymously donated to research after informed written consent from donors in concordance with French legislation (94–654 and 08–400) and with prior approval of the protocol (to M.V.) from the Necker ethical review committee. All mice used in this study were housed under specific pathogen-free conditions at the mouse genetics engineering center (C.I.G.M.) of the Pasteur Institute, Paris, under authorization number A75-15-09 from the Paris Departmental Directorate for the Protection of Populations and handled in accordance with French and European directives.

### Chromatin immunoprecipitation

ChIP was carried out as previously described, starting from nuclear isolation [77], using eleven microdissected and flash-frozen cardiac tubes from human embryos at Carnegie stages (CS) 14–15 [78]. An anti-ISL1 (10 µL, Santa Cruz Sc-23590X) or an anti-GFP antibody as negative control (10 µL, Abcam ab1218), were used per 10 µg of sonicated chromatin. Immunoprecipitated DNA was analysed by end-point PCR (primers, Supplementary Table S1).

### Expression studies

*ISL1* and *GATA4 in situ* hybridizations were performed using transverse sections of normal human embryos from CS12 to 15. Tissue fixation, sectioning, and *in situ* hybridization were carried out as previously described [79]. Total RNA was extracted from pooled whole hearts at individual stages from CS13 to CS16 and RT-PCR was carried out using the GeneAmp kit (Roche), with 500 ng total RNA input for first strand synthesis (primers, Supplementary Table S1).

### Expression constructs and electrophoretic mobility shift assays (EMSA)

Human TBX20 and ISL1 expression vectors were generated. Full-length *TBX20* cDNA and a fragment of *ISL1* cDNA with the N-terminal 142 amino acids removed [80] were inserted into the multiple cloning site of pcDNA3.1C (Invitrogen). Full-length human *GATA4* cDNA was purchased from GenScript (GN026113). HeLa cells were transfected with these constructs, and nuclear protein extracts were made using standard protocols. The LightShift Chemiluminescent EMSA Kit (Pierce) was used as specified. Primers are listed in Supplementary Table S1.

### Transactivation assays and reporter constructs

For the *FGF10* reporter construct (LUC-*FGF10*-Int1), 1047 bp of the *FGF10* first intron (NCBI36/hg18 chromosome 5:44421556–44422602) were subcloned into the BamHI site 3′ to luc+ in pGL3 (Promega). Mouse 10T1/2 cells [81] in DMEM/10% fetal calf serum were transfected with FuGene HD (Roche). Cells were harvested and lysed 24 h after transfection. Firefly and Renilla luciferase activities were measured on a Berthold Centro LB960 using the Dual-Luciferase Reporter assay system (Promega). Firefly luciferase activity was normalized to the Renilla luciferase internal control, pRL-CMV (Promega). Experiments were repeated in triplicate in three independent assays.

### Transgenesis

The same 1047 bp *FGF10*-Int1 fragment as in the transactivation assays was subcloned into the BamHI site of the pSKT-TK-nLacZ plasmid [46] and orientation verified by capillary sequencing with a standard T3 primer. The plasmid was linearized with SalI for injection at 2 ng/mL into mouse blastocysts. β-galactosidase-containing cells that had transcribed the reporter plasmid were stained in whole mount by the catalysis of the X-gal (5-bromo-4-chloro-3-indolyl β-D-galactopyranoside) substrate.

## Supporting Information

Figure S1 Composite image of embryonic hearts at stages ranging from the beginning of the fourth to the ninth week of human gestation (upper left to lower right, Carnegie stages 10–23). Rostral to top. Congenital heart and great vessel malformations arise during this time window when molecular signaling between cardiac progenitors and their environment is impaired.
(TIF)

Table S1 Primer sequences for PCR and EMSA.
(DOC)
