# Supplementary figures and images for: ISL1 Directly Regulates FGF10 Transcription during Human Cardiac Outflow Formation
Source: PLoS One. 2012 Jan 27;7(1):e30677. doi: 10.1371/journal.pone.0030677 (PMC3267757; doi:10.1371/journal.pone.0030677)

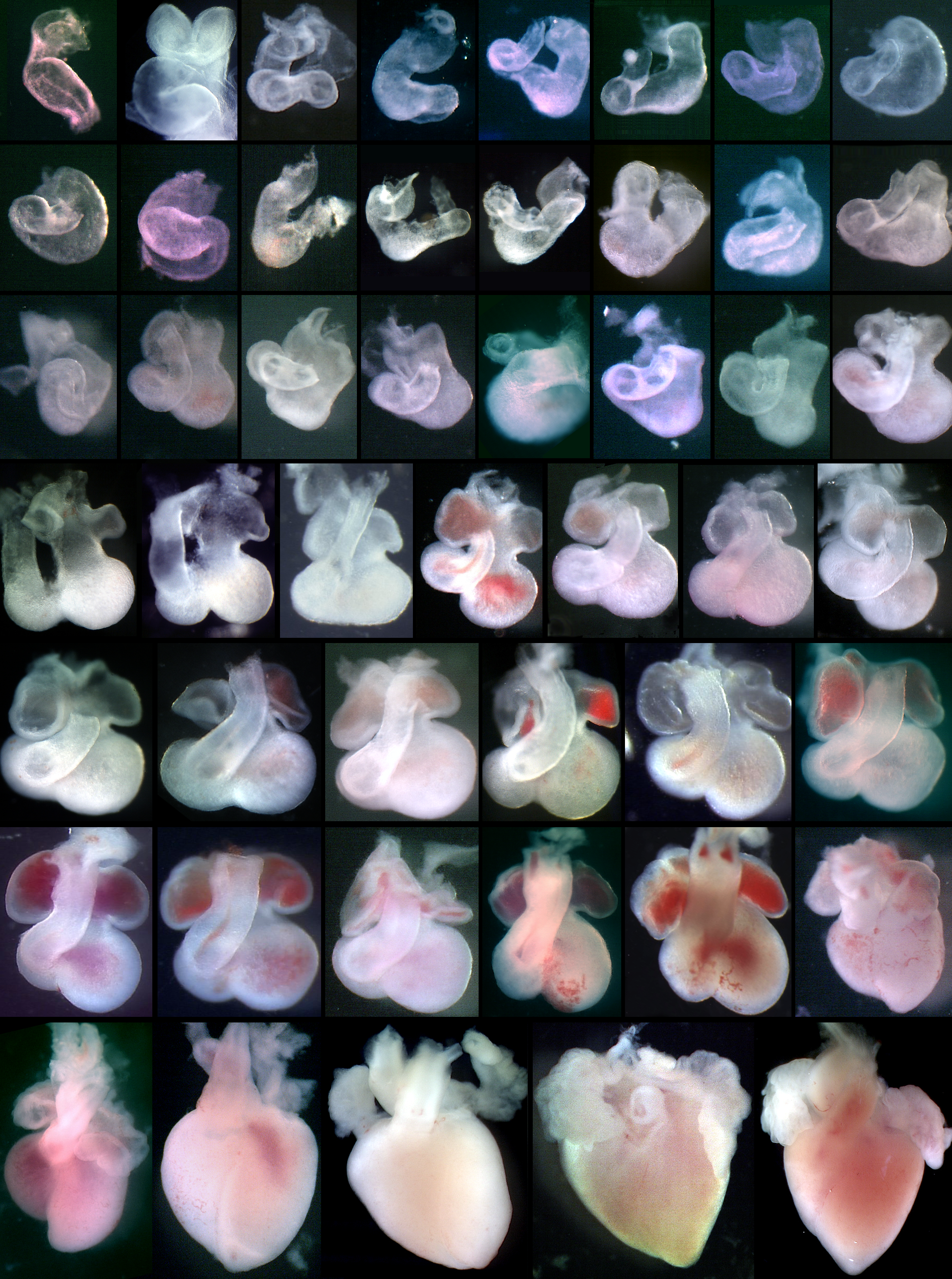

Supplement: Figure S1 — Composite image of embryonic hearts at stages ranging from the beginning of the fourth to the ninth week of human gestation (upper left to lower right, Carnegie stages 10–23). Rostral to top. Congenital heart and great vessel malformations arise during this time window when molecular signaling between cardiac progenitors and their environment is impaired. (TIF) [file pone.0030677.s001.tif]
