# Supplementary material for: ISL1 Directly Regulates FGF10 Transcription during Human Cardiac Outflow Formation
Source: PLoS One. 2012 Jan 27;7(1):e30677. doi: 10.1371/journal.pone.0030677 (PMC3267757; doi:10.1371/journal.pone.0030677)
Supplement: Table S1 — Primer sequences for PCR and EMSA. (DOC) [file pone.0030677.s002.doc]

| **For *in situ* hybridization** | |
| --- | --- |
| hISL1-T7-F | TAATACGACTCACTATAGGGAGAGGTTGTACGGGATCAAATGC |
| hISL1-R | GCCCGTCATCTCTACCAGTT |
| hISL1-T7-R | TAATACGACTCACTATAGGGAGAGCCCGTCATCTCTACCAGTT |
| hISL1-F | GGTTGTACGGGATCAAATGC |
| hGATA4-T7-F | TAATACGACTCACTATAGGGAGATCTTGCAATGCGGAAAGAG |
| hGATA4-R | CAGTGATTATGTCCCCGTGA |
| hGATA4-T7-R | TAATACGACTCACTATAGGGAGACAGTGATTATGTCCCCGTGA |
| hGATA4-F | TCTTGCAATGCGGAAAGAG |
| **For RT-PCR** | |
| hISL1-F | GGTTGTACGGGATCAAATGC |
| hISL1-R | GCCCGTCATCTCTACCAGTT |
| hGATA4-F | TCTTGCAATGCGGAAAGAG |
| hGATA4-R | CAGTGATTATGTCCCCGTGA |
| hGATA5-F | CCTGCGGCCTCTACATGA |
| hGATA5-R | AGGCTCGAACTTGAACTCCA |
| hGATA6-F | GTGCCCAGACCACTTGCTAT |
| hGATA6-R | GCGAGACTGACGCCTATGTA |
| hFGF10-F | TGCTGCTTTTTGTTGCTGTT |
| hFGF10-R | CATTTGCCTCCCATTATGCT |
| hACTB-F | ATTGGCAATGAGCGGTTCCGC |
| hACTB-R | TCCTGCTTGCTGATCCACATC |
| hNUP54-F | ACGCTGTTGGGAGATGAGAG |
| hNUP54-R | GCTTGTTCAAAATGGGCATA |
| hJAKMIP3-F | AGCTGCTGTCAGAGGAGGAG |
| hJAKMIP3-R | AAAGGGTCCGATTCAATGTG |
| hBMP2-F | GTTCGGCCTGAAACAGAGAC |
| hBMP2-R | AATTCGGTGATGGAAACTGC |
| hDOK5-F | TCAATGACATCAGCCTTGGA |
| hDOK5-R | AGGCAGCAGAGTGGACTTTC |
| hMAP4K4-F | AGGCCAGAGGTTGAAAGTGA |
| hMAP4K4-R | TGACCAGTTTCCACAGATCG |
| hMYO1D-F | CCCTTCTTTACCGGACTGTG |
| hMYO1D-R | GCTGCTGCAGTTTCTCATTG |
| hUNC45B-F | GCCATTCATGACAACTCACG |
| hUNC45B-R | TAGTGCCACCATCATCTCCA |
| hROCK1-F | CAACAACGGTTAGAACAAGAGG |
| hROCK1-R | TTGTCTGCCTCAAATGCTTG |
| hPDE3A-F | TCATCCAGGAAGGACTAATGC |
| hPDE3A-R | GGACCATTGATATCAGCCAAC |
| **For ChIP** | |
| hISL1-Prom-F | CCTCCCACCCAACGTTTTTA |
| hISL1-Prom-R | CGAGTGGCTGGTGGGTAG |
| hFGF10-Prom-F | TTTGTTCACCGTGCTGTCAT |
| hFGF10-Prom-R | GATGCAAGGCAAGGAGAGAG |
| *FGF10*-Pr1-F | gggagccaatttcattttca |
| *FGF10*-Pr1-R | ggagcactgtgacaaaa |
| *FGF10*-Pr2-F | ttcttttctgtgcagcctttc |
| *FGF10*-Pr2-R | tgtccttttcaatcctagcaaa |
| *FGF10*-Int1-F | ggaaaaggaattgacactcttca |
| *FGF10*-Int1-R | ggaggggttcactctgctaa |
| **For EMSA** | |
| InsulinS (+ control) | gcccttgttaataatctaattaccctag |
| InsulinAS | ctagggtaattagattattaacaagggc |
| FGF10-int1-S | tagacaatatcttaatgataccatgtag |
| FGF10-int1-AS | ctacatggtatcattaagatattgtcta |
| FGF10-int1mut-S | tagacaatatcttagctataccatgtag |
| FGF10-int1mut-AS | ctacatggtatagctaagatattgtcta |
| GATA4-S | gtagcagcatttagattacctggccacatg |
| GATA4-AS | catgtggccaggtaatctaaatgctgctac |
| GATA4mut-S | gtagcagcatttaccatacctggccacatg |
| GATA4mut-AS | catgtggccaggtggtataaatgctgctac |
